# Recurrent Glomerulonephritis after Renal Transplantation: The Clinical Problem

**DOI:** 10.3390/ijms21175954

**Published:** 2020-08-19

**Authors:** Barbara Infante, Michele Rossini, Serena Leo, Dario Troise, Giuseppe Stefano Netti, Elena Ranieri, Loreto Gesualdo, Giuseppe Castellano, Giovanni Stallone

**Affiliations:** 1 Nephrology, Dialysis and Transplantation Unit, Department of Medical and Surgical Sciences, University of Foggia, Viale Pinto Luigi 251, 71122 Foggia, Italy; barbarinf@libero.it (B.I.); sereleo1989@libero.it (S.L.); dario.troise@gmail.com (D.T.); giovanni.stallone@unifg.it (G.S.); 2 Clinical Pathology Unit and Center of Molecular Medicine, Department of Medical and Surgical Sciences, University of Foggia, Viale Luigi Pinto, 71122 Foggia, Italy; michelerossini@libero.it (M.R.); giuseppestefano.netti@unifg.it (G.S.N.); elena.ranieri@unifg.it (E.R.); 3 Nephrology, Dialysis and Transplantation Unit, Department of Emergency and Organ Transplantation, University of Bari, 70124 Bari, Italy; loreto.gesualdo@uniba.it

**Keywords:** kidney transplant, recurrent glomerulonephritis, pathology, bimolecular mechanisms

## Abstract

Glomerulonephritis (GN) continues to be one of the main causes of end-stage kidney disease (ESKD) with an incidence rating from 10.5% to 38.2%. Therefore, recurrent GN, previously considered to be a minor contributor to graft loss, is the third most common cause of graft failure 10 years after renal transplantation. However, the incidence, pathogenesis, and natural course of recurrences are still not completely understood. This review focuses on the most frequent diseases that recur after renal transplantation, analyzing rate of recurrence, epidemiology and risk factors, pathogenesis and bimolecular mechanisms, clinical presentation, diagnosis, and therapy, taking into consideration the limited data available in the literature. First of all, the risk for recurrence depends on the type of glomerulonephritis. For example, recipient patients with anti-glomerular basement membrane (GBM) disease present recurrence rarely, but often exhibit rapid graft loss. On the other hand, recipient patients with C3 glomerulonephritis present recurrence in more than 50% of cases, although the disease is generally slowly progressive. It should not be forgotten that every condition that can lead to chronic graft dysfunction should be considered in the differential diagnosis of recurrence. Therefore, a complete workup of renal biopsy, including light, immunofluorescence and electron microscopy study, is essential to provide the diagnosis, excluding alternative diagnosis that may require different treatment. We will examine in detail the biomolecular mechanisms of both native and transplanted kidney diseases, monitoring the risk of recurrence and optimizing the available treatment options.

## 1. Introduction

Recurrent glomerulonephritis (GN) and the onset of new GN (de novo GN) after renal transplantation are common problems and many cases have been reported since the early days of transplantation [1]. Indeed, although transplantation may restart kidney function, often the cause of the original kidney disease remains unknown [2].

GN continues to be one of the main causes of end-stage kidney disease (ESKD) worldwide, with an incidence rating from 10.5% to 38.2% and a prevalence rating from 17.6% to 53.5% [3]. Though recurrent GN was previously considered to be a minor contributor to graft loss, several studies have shown that about 10–20% of patients with a glomerular disease develop recurrence after renal transplantation and 50% of them show a graft loss on long term follow up [4,5]. On the other hand, propensity for GN to recur seems to depend on the graft survival increase [2].

Briganti et al. took into account 1505 patients with both native and graft biopsies, evidencing that recurrent disease is the third most common cause of graft failure 10 years after renal transplantation, whereas death with a functioning graft and chronic rejection (CR) are the first and the second cause, respectively, and acute rejection the fourth [4].

However, GN is characterized by heterogeneous histological subtypes, causes, and clinical manifestations, resulting in different prognosis after kidney transplantation [6]. Therefore, clinicians should provide adequate information on the risks of post-transplant recurrence, assessing the medical adequacy of patients with GN for renal transplantation [7,8].

Virtually every GN may recur after transplantation, however the impact and consequences of recurrence can be very different. For example, recipient patients with anti-glomerular basement membrane (GBM) disease present recurrence rarely, but often exhibit rapid graft loss. On the other hand, recipient patients with C3 glomerulonephritis present recurrence in more than 50% of cases, although the disease is generally slowly progressive with a mean graft survival of 6.4 years from transplantation [3,9].

According to clinical criteria, recurrent and de novo GN, can be described as *True recurrence* (native and recurrent disease are the same at biopsy), *Transplant GN* with unknown primary disease, de novo *GN* (occurrence of new disease in the graft). Recurrence can occur for both primary and secondary GN. *Recurrence of primary GN*: recurrent focal and segmental glomerulosclerosis (FSGS), membrano-proliferative GN (MPGN), IgA nephropathy (IgAN), Henoch-Schonlein purpura, Membranous Nephropathy (MN). *Recurrence of secondary GN*: systemic lupus erythematosus (SLE), hemolytic uremic syndrome/thrombotic thrombocytopenic purpura (HUS-TTP), small vessel vasculitis, anti-glomerular basement membrane (anti-GBM) disease. We can also observe *recurrence of metabolic or systemic disease* such as diabetic nephropathy, oxalosis, amyloidosis, Fabry disease, cystinosis, fibrillary GN; de novo GN more frequently encountered are anti-GBM disease in patients with Alport syndrome, MN, FSGS [1].

Diagnosis and management of recurrence are fundamental objectives aimed at optimizing graft survival and analyzing the pathogenesis of native kidney disease [10].

The purpose of this review is to describe the most frequent diseases that recur after renal transplantation, analyzing rate of recurrence, epidemiology and risk factors, pathogenesis and bimolecular mechanisms, clinical presentation, diagnosis and therapy, taking into consideration the limited data available in the literature.

## 2. Epidemiology and Risk Factors

**Figure 1:**
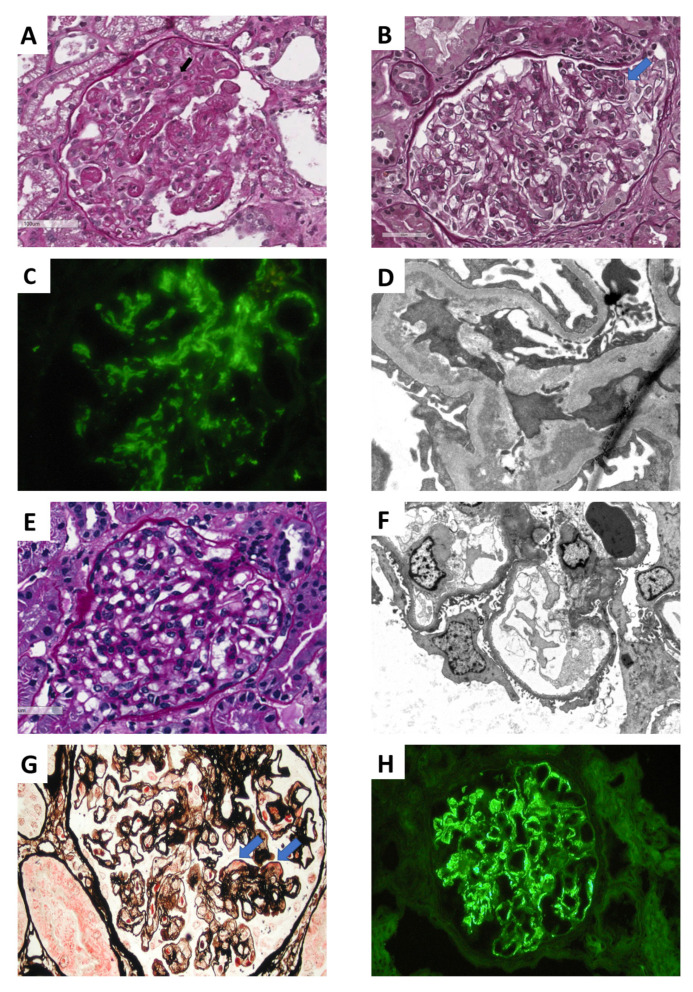
Glomerular fibrin and platelet thrombi in a patient with *recurrent aHUS* (PAS) (**A**). In a patient with *early recurrent C3 glomerulonephritis* the light microscopy (**B**) shows mild mesangial proliferation with segmental endocapillary hypercellularity (arrows, PAS staining). In this patient immunofluorescence shows only C3 mesangial staining (**C**) with electron dense deposits in mesangial and subendothelial spaces by electron microscopy (**D**). In a patient with *early recurrent focal segmental glomerulosclerosis*, glomeruli may look normal by light microscopy (**E**, PAS staining). Podocyte injury is revealed by electron microscopy showing diffuse foot processes effacement (**F**). In a patient with *recurrent IgAN* the light microscopy picture (**G**) shows a glomerulus with global membranoproliferative pattern of injury with several aspects of glomerular basement membranes double contours as well mesangial and capillary wall eosinophilic deposits (arrow) (Jones silver stain). In this patient immunofluorescence microscopy revealed mesangial and glomerular capillary wall deposition of IgA (**H**), C4d was negative.

Diagnosis of “true” recurrence requires renal biopsy of both native and transplanted kidneys. However, biopsy of native kidney is not always available [11]. In addition to this, many patients with post-transplantation deterioration of renal function and proteinuria are misclassified as chronic rejection and are not biopsied or biopsy is not done with recurrence in mind, so immunofluorescence studies and electron microscopic examinations may not be undertaken [2] (Figure 1). Moreover, renal biopsy is often unable to identify the cause of graft loss as a result of advanced scarring of renal tissue [12]. Thus, the incidence of recurrence and the reported allograft loss rates attributed to it, varying between 7% and 55% internationally, are probably underestimated [8,13].

Golgert et al. have analyzed epidemiology of native kidney disease and recurrent GN after renal transplantation using the data of several registries related to different countries. Their study showed that the prevalence rates of native kidney GN and recurrent GN are higher in children and white patients, due to a low prevalence of diabetes in children and a higher prevalence of hypertensive nephrosclerosis and diabetic nephropathy in black patients [1].

Several factors may influence the risk for recurrence. First of all, it depends on the type of glomerulonephritis. For example, lupus nephritis recurs in fewer than 10% of cases and graft loss is uncommon, in contrast C3 glomerulopathy recurs in more than 80% of patients and graft loss is frequent [14]. Time since transplantation may be related to the duration of the graft exposure to the nephritogenic factors responsible for GN [15]. The recipients of human leukocyte antigen (HLA)-identical transplants promote graft survival with an increased risk of recurrence [16].

Jiang et al. conducted a study showing that recurrent GN also depends on male gender, early age, duration on dialysis less than 5 years before transplant; this study confirm that there is no evidence for an increased risk of recurrence in a subsequent graft except for FSGS [17,18]; on the contrary, Ohmacht et al. highlight higher risks in IgAN for second graft [19].

Several strategies to reduce the risk of recurrence have been reported. Bilateral native nephrectomy to eliminate persistent antigenic stimulation demonstrates no benefit [20]. Other strategies have included induction of disease remission before transplantation and longest time on dialysis pre-transplantation. These issues are discussed within real benefits except for the case of anti-GBM disease, where a negative serological test for at least 6 months before kidney transplantation might be associated with low risk of recurrence [4].

## 3. Clinical Features and Differential Diagnosis

Clinical features of recurrence are often the same of native disease: proteinuria, hematuria, deterioration in renal function. At the time of recurrence renal function may be reduced or normal with a progressive graft loss extremely variable [2]. Nevertheless, even chronic rejection may manifest with progressive deterioration of kidney function, proteinuria, and hypertension, potentially being clinically indistinguishable from recurrence [21].

It is known that some recurrent GN may predispose the graft to rejection, for example FSGS, and vice versa [22]. Furthermore, recurrence may coexist with chronic allograft rejection or calcineurin-inhibitor (CNI) toxicity. Every condition that can lead to chronic graft dysfunction should be considered in the differential diagnosis of recurrence. Renal biopsy is essential, it can provide the diagnosis, excluding alternative diagnosis that may require different treatment, and provides some important information on the possibility of a future re-transplantation [23].

A complete workup of renal biopsy, including light, immunofluorescence, and electron microscopy study, allow us to distinguish recurrent GN from rejection, drug toxicities, infection, etc. The presence of tubulitis (the presence of mononuclear cells in the basolateral aspect of the renal tubule epithelium) and interstitial inflammation suggest T Cell-Mediated Rejection (TCMR) [24,25]. The presence of striped interstitial fibrosis, tubular atrophy, and nodular arteriolar hyalinosis are suggestive of CNI Nephrotoxicity [21]. These changes are due to the increased level of TGF-β that promotes the development of fibrosis and epithelial–mesenchymal transition in which mesenchymal or epithelial cells can turn into fibroblasts, leading to fibrosis [26].

Conversely, histologic features characteristic of active antibody-mediated rejection (AMR) are glomerulitis (a form of micro-vascular inflammation), peritubular capillaritis (inflammation within peritubular capillaries), intimal arteritis (inflammation within the arterial intima), with or without C4d positivity, with or without evidence of circulating antibodies reactive to the donor, HLA or other endothelial antigens [24,27,28]. Chronic active AMR rejection is characterized at the glomerular level by “transplant glomerulopathy”, changes that include glomerular basement membrane (GBM) reduplication with or without cellular proliferation, which may be indistinguishable, by light microscopy alone, from a membrano-proliferative glomerulonephritis (MPGN). Indeed, GBM duplication is the hallmark of chronic endothelial injury that can be caused by a variety of insults to the allograft glomerulus, including donor specific antibodies in AMR, immune complexes in recurrent or de novo GN, complement mediated in atypical HUS thrombotic microangiopathy [29].

For this reason, the lesions identified in light microscopy need to be interpreted along with immunofluorescence studies (presence of immunoglobulin and complement deposits at the glomerular level, C4d in peritubular capillaries), electron microscopy that may clarify the structure of basement membrane and location of deposits and a complete clinical history and laboratory information (e.g., DSA status, complement levels, etc.) [23,30].

## 4. Primary GN IgA Nephropathy (IgAN)

IgAN is the most prevalent form of primary glomerulonephritis globally, and a common cause of end-stage kidney disease (ESKD) [3,31]. Commonly, patients with IgAN are younger, less affected by comorbidities (e.g., diabetes and/or cardio-vascular disease) than older patients with ESKD, and consequently are more frequently suitable for transplantation [32].

Several studies demonstrated that IgAN recurs after renal transplantation in a percentage varying from 9% to 61%, depending on differences in follow-up duration and biopsy policies, and that recurrence leads to graft dysfunction in approximately 13% of patients and to graft loss in nearly 5% of cases [33,34,35].

The pathogenesis of IgAN is not completely understood [36]. There appears to be a genetic predisposition in patients that develop this kind of nephropathy, with specific HLA types associated with high serum IgA concentration [37,38].

Several studies have shown that in patients with IgAN there is a defect of beta 1-3 galactose-transferase that increases the production of IgA1 with galactose-deficiency in the hinge-region, called galactose-deficient IgA1 (Gd-IgA1) [39]. This defect leads to an impaired structure of IgA1, synthesis of antibodies directed against galactose-deficient IgA1 (IgG), binding of the galactose-deficient IgA1 by the anti-glycan/glycopeptides antibodies, thus inducing the formation of circulating immune complexes and the accumulation of these complexes in the glomerular mesangium. On the other hand, these polymeric IgA1 have an increased tendency for the formation of macro aggregates, which seep into the kidney, leading to the formation of in situ immune complexes [40].

The deposition of immune complex, that causes mesangial cells proliferation, matrix expansion, inducing the synthesis of numerous growth factors and cytokines attracting inflammatory cells on site, is possible due to the presence of IgA receptors in the kidney. Indeed, IgA are bound by IgA fragment crystallizable alpha receptor or CD89 [41].

Several studies have demonstrated the presence of a molecule, CD71, a transferrin receptor, that binds polymeric IgA1 and is overexpressed on mesangial cells in patients with IgA nephropathy. This abnormal CD71 expression mediates the deposition of circulating Gd-IgA1-IgG complexes in the mesangium and stimulates the mesangial expression of transglutaminase 2. Transglutaminase 2 contributes to an amplification loop of IgA1-CD89 deposition in the glomeruli [42]. In patients with IgAN, investigators found an increased expression of these receptors. Moreover, their soluble form found in the urine could be considered a potential marker for monitoring the progression of renal damage [43]. The link between IgA and CD71 causes the secretion of pro-inflammatory cytokine through the activation of the protein kinase (MAPK) pathway [44].

In addition, these Gd-IgA1-IgG have been proven capable of activating an alternative complement pathway, because C3 is frequently involved in the formation of circulating immune deposits inducing a secretory phenotype of the mesangial cells [45,46].

On the basis of these described mechanisms IgAN is classified as an autoimmune disease [40]. Recent studies have focused on the B cell activation factor of tumor necrosis factor superfamily 13 (TNFSF13), also known as a proliferation-inducing ligand (APRIL) which appears to be reversed in increasing of IgA secretion and in the production of anti-glycan antibody [40,47]. APRIL is secreted from antigen-presenting cells such as macrophages or dendritic cells and binds the receptors on B and T cells, therefore this factor is involved in the innate and adaptive immunity [48]. Therefore, high serum APRIL/TNFSF13 levels in patients with IgAN could predict the progression of renal disease [47].

Analysis of risk factors for IgAN recurrence have not provided consistent results. However, the rate of recurrence seems to be time-dependent, progressively increasing after transplantation [49]. Younger age at renal transplantation, recipients of zero-HLA mismatched live-related donor kidney, steroid-avoidance or early steroid-withdrawal immunosuppressive regimens, male gender, rapidly progressive course of the original disease before transplantation, degree of proteinuria, HLA-B35/DR4, and higher levels of circulating Gd-IgA1 and IgA-IgG immune complexes are all probably associated with a higher risk of recurrence [50,51]. Several molecules, such as soluble CD89, may be related to an increased risk of disease progression and of recurrence after transplantation [52].

Recurrence more often occurs 3 years after transplantation, reducing the graft survival only in the long term [53].

Many studies have found that the rate of recurrence increases in patients with a living related donor, and even with increased serum levels of IgA 6 months post-transplants [54]. Berthelot et al. demonstrated that low levels of CD89 (a leukocyte cell surface receptor for IgA) may be associated with a more aggressive disease in the native kidney, growing the risk of recurrence after transplantation [52].

Previous studies report that there is a grown risk of recurrence of IgAN in a subsequent graft [55]. Conversely, recent studies suggested that there is no increased risk of recurrence on patients who lost the first graft for recurrence [18].

Furthermore, Avasare et al. hypothesized a correlation for an adverse outcome between the number of crescents in the native biopsy and both the renal native survival and the risk of recurrence post transplantation, increasing the probability of allograft rejection [56].

Clinical manifestations of IgAN range from macroscopic hematuria with or without acute kidney dysfunction during infective events of respiratory or gastrointestinal tracts, to microscopic hematuria isolated or associated with proteinuria and hypertension [57]. Clinical presentation of recurrence is similar to primary IgAN with microscopic hematuria, proteinuria, slow decline kidney function, following a benign course initially [58]. Instead, a not benign course has been reported with increasing long-term data [59]. Nevertheless, macroscopic and often microscopic hematuria, the hallmarks of IgAN, are rarely present in recurrence at the time of the diagnosis, more often isolated proteinuria is the only sign [34].

However, many patients with recurrent IgA do not have clinical signs and the diagnosis can be histological only with mesangial IgA deposits with or without mesangial proliferation [36,60]. In a minority of cases cellular, fibro-cellular, or fibrous crescents are described at the graft biopsy and they are associated with a significantly worse graft survival [61].

Oxford Classification has well defined the clinical implications of histological signs found at the biopsy in the native kidney [62]. Regardless of clinical signs, the degree of severity of the disease depends on five histological features: degree of mesangial hypercellularity, segmental glomerulosclerosis, endocapillary hypercellularity, tubular atrophy and interstitial fibrosis, and cellular and/or fibrocellular crescents (MEST-C) [63]. Oxford classification criteria have been successfully applied to recurrent IgAN and provide useful prognostic information for graft failure [64,65].

To date, there are no specific therapies for recurrent IgAN yet. Recently, the Kidney Disease Improving Global Outcomes (KDIGO) Transplant Guidelines has clarified the management of patients affected by recurrent IgAN, recommending the reduction of proteinuria and blood pressure control [66]. Only one study demonstrated that the graft survival is increased by the use of angiotensin converting enzyme inhibitors [67].

In the last few years there has been much discussion about how much induction therapy can affect the recurrence of this nephropathy. Bertoux et al. showed that the incidence of recurrence of IgAN at the tenth post transplantation year is about 36% in the whole population, but only 9% in patients with anti-thymocyte globulin (ATG) induction therapy, in comparison with 41% in patients without induction therapy [68]. Another study shows a reduced incidence of recurrence of IgAN in patients with ATG induction therapy in comparison with Basiliximab and Alemtuzumab induced patients [69].

For the immunosuppressive maintenance therapy, Mulayet al. did not find any association between the immunosuppressive drugs and the rate of graft failure caused by recurrence [70]. Nevertheless, in literature, steroid withdrawal has been associated with increased rates of recurrence and the combination of mycophenolate and tacrolimus may also be protective for graft survival [71].

## 5. Focal and Segmental Glomerulosclerosis (FSGS)

### 5.1. Pathogenesis

The pathogenesis of recurrent FSGS is a hotly debated topic in literature. FSGS is characterized by the histopathological finding of segmental sclerosis of the capillary tuft of the glomerulus due to podocyte injury/disease. Podocytes are highly specialized cells. Their functions are as follows: support of glomerular capillaries, glomerular basement membrane (GBM) protein synthesis, adjustment of glomerular permeability. These complex functions depend on the cyto-architecture of the podocyte that is expressed by the progressive acquisition of some cellular markers: Wilms’ tumor protein (WT-1), Common Acute Lymphoblastic Leukemia Antigen (CALLA), C3b receptor, Glomerular Epithelial Cell Protein-1 (GLEPP-1), podocalyxin, and sinaptopodin [72]. Damaged podocytes are unable to maintain their phenotypic differentiation and undergo various morphological and structural changes: cell hypertrophy, diffuse effacement of foot processes, formation of pseudocysts, detachment from GBM and, sometimes, hyperplasia. Parietal epithelial cells adhere to the GBM, detach from each other, causing direct contact between GBM and parietal basal membrane (PBM), so-called adhesion which is the early sign of a segmentally sclerotic lesion [73].

In the last years, experts have focused on the inter-podocyte connections. The slit-diaphragm consists of structural proteins, such as nephrin and P-cadherin that are anchored by a protein called podocin. From literature data, it has been hypothesized that the permeability factor may lead to the loss of nephrin and to the reduction of the expression of podocin [74]. Indeed, mutation of the gene NPHS2 which encodes for podocin, is associated with familiar and sporadic FSGS and the rate risk of recurrence in patients with this mutation is around 8%, contrary to the belief that familiar forms of FSGS do not recur [75]. In particular, suPAR seems to alter podocyte cytoskeleton and podocyte attachment with activation of beta 3-integrin and STAT1 in vascular smooth muscle cells through a PDGF receptor [76]. However, CLCF-1 performs its action through a complex receptor composed of ciliary neutrotrophic factor receptor (CNTFR), leukemia inhibitory factor receptor (LIFR), glycoprotein 130 (gp130). The complex receptor-ligand alters podocyte actin cytoskeleton activating the JAK/STAT (janus kinase/signal transducer and activator of transcription) pathway, that when inhibited by aspecific monoclonal antibody in rats reduced glomerular permeability [77].

An important pathogenic sign is the hyperplasia of the podocytes. These cells generally are incapable of proliferating. Podocyte quiescence requires the presence of the Cip/Kip family of cyclin-dependent kinase inhibitors p27 and p57. Nevertheless, in FSGS disease differential expression of cyclin-dependent kinase inhibitors has been highlighted [78].

The normal mature podocyte does not express proliferation markers (PCNA and Ki-67). However hyperplastic cells are characterized by the expression of nuclear proliferation markers (PCNA and Ki-67), the loss of normal podocyte markers (WT1, CALLA, GLEPP1 etc.), and positive immune-staining for macrophage and cytokeratin markers (CD 68 and AE1/AE3 CK) [79].

Charba et al. have posed the problem that patients with FSGS may produce anti-actin and anti-nephrin auto-antibodies that may cooperate to the onset of recurrence. They injected antibodies directed against the protein tyrosine phosphatase Receptor type O (PTRO), that regulates nephrin in the filtration barrier, increasing proteinuria after transplantation [80].

Nonetheless, recent literature demonstrates that the hyperplastic cells do not express markers typical of podocyte. Indeed, they express CK8, PAN, cadherin, claudin-1, and PAX-2 which are usually markers of parietal cells [81]. This probably confirms that proliferated epithelial cells originate from parietal epithelial cells [82]. Therefore, podocyte injury and subsequent podocyte or parietal epithelial cell hyperplasia is at the base of the pathogenic mechanism of FSGS and recurrence [83].

Clinically it is distinguished by proteinuria, generally in the nephrotic range [84,85]. FSGS can be idiopathic, secondary and reactive, or poorly adapted. The primary form includes all those in which the cause is unknown; the secondary form includes a variety of etiologies, such as genetic, viral (e.g., HIV, parvovirus B19, cytomegalovirus, EBV), drug-induced (e.g., heroin, lithium, interferon, calcineurin inhibitors); the reactive form represents the final histological lesions that are common to any progressive renal damage [86,87]. Typically, only primary FSGS recurs after renal transplantation [88]. Indeed, Maas et al. demonstrate that recurrence only occurs in the idiopathic form and not in the genetic or secondary forms [89]. In primary form, about 40–60% of patients develop ESRD within 20 years and generally it recurs in approximately 30–50% of patients after renal transplantation, more often in the children, increasing graft failure rate [90,91,92]. However, a de novo FSGS can also occur in the recipient when his original disease is not FSGS [93]. Interestingly, recurrent FSGS can lead to early graft failure from 12% to 27% of cases [94].

### 5.2. Clinical Presentation of FSGS Recurrence

There are two clinical manifestations of recurrent FSGS: an early recurrence characterized by a massive proteinuria within 48–72 h after transplantation and a late recurrence, characterized by a progressive development of the nephrotic syndrome within months or years after surgery [95]. In the case of early recurrence, immediately or few days after surgery, histological lesions by light microscopy are generally not present and segmentally sclerotic lesions may occur only later [96,97]. In fact, the diffuse effacement of foot processes by electron microscopy is the only initial histologic finding of early recurrent FSGS if ultrastructural examination is performed. In transplanted patients the differential diagnosis between recurrence and FSGS caused by Calcineurin-inhibitors (CNI) or other causes such as obesity and hypertension, is difficult, especially in the advanced phase, but in the latter case the diffuse effacement of foot processes is less obvious.

The frequent occurrence of proteinuria within a few hours or days after transplantation suggests that podocyte injury is probably caused by a circulating permeability factor [98]. Numerous observations seem to confirm this hypothesis as, for example, frequently occurring proteinuria in patients with FSGS undergoing transplantation or plasmapheresis and immune absorption who have been shown to be effective in reducing proteinuria [99]. The studies show that this circulating factor, which has an apparent molecular mass of about 50 KDa, binds to protein A and may be part of a complex with immunoglobulins [100]. A recent study isolates some proteins such as suPAR, cardiotrophin-like cytokine-1 (CLCF-1), apolipoprotein A-1 [101].

The average time of onset of recurrence is 2 weeks in children and 7.5 months in adult patients [102]. However, often these patients may have an early recurrence with a proteinuria usually in a nephrotic range, within a few hours of surgery [74]. Besides, in patients who have had recurrent FSGS in the first transplantation, the risk of recurrence in the second graft is exponentially greater [103].

### 5.3. Risk Factors and Biomarkers of FSGS Recurrence

Several risk factors have been described to be associated with FSGS recurrence, such as younger age of the recipients [104], rapid progression to ESRD [105], mesangial proliferation in the native kidney biopsy (reflecting a more severe form of disease) and steroid resistance [106], older donor [107], pre-transplant bilateral nephrectomy (native kidney seems to be absorber of permeability factors) [108], and recurrence of FSGS in a previous allograft [102]. Finally, ethnicity also influences the incidence of recurrence that is higher in white than in non-Caucasian patients [109]; a lower rate of recurrent disease is described for African American patients compared to other races [94]. Furthermore, the duration of dialysis and the type of post-transplant immunosuppression seem to be risk factors of recurrence [110]. A recent study by TANGO project group demonstrated that idiopathic FSGS recurs post-transplant in one third of cases and is associated with a five-fold higher risk of graft loss; moreover, the authors demonstrated that a response to treatment was associated with significantly better outcomes achieved in only half of the cases analyzed [75].

The histologic type of FSGS seems not to provide correlation with the risk of recurrence. Swaminathan et al. show that collapsing FSGS has a low risk of recurrence compared to non-collapsing types [111]. A recent review of United Network for Organ Sharing (UNOS) data over the 20-year period of 1988–2008 indicates that donor type does not alter recurrence risk [112]. On the other hand, Abbott et al. demonstrated an higher risk of recurrence in living-related donation compared with deceased donor, particularly in the pediatric recipients [109].

Data available in literature on risk of recurrence associated with induction therapy are inconclusive. Raafat et al. point out that use of anti-thymocyte globulin (ATG) is associated with a higher risk of recurrence [113]. Conversely, Pascual et al. demonstrate that induction therapy with ATG is related to a minor risk of recurrence [114].

Several molecules may be biomarkers to define the risk of recurrence. Increased levels of Soluble Urokinase-type Plasminogen Activator Receptor (suPAR) before transplantation seem to be related with a higher risk of recurrence [115]. Indeed, the use of plasmapheresis and immunoadsorption reduces suPAR levels with remission of proteinuria [116]. Nevertheless, higher suPAR levels have been registered also in various cancers and in other inflammatory disease, as pneumonia, malaria, tuberculosis, HIV, sepsis [117]. Delville et al. identify seven antibodies that may be related with recurrent FSGS: CD40 (correlated with a greater risk of recurrence), PTPRO, CGB5, FAS, P2RY11, SNRPB2, and APOL2 [118].

### 5.4. Treatment of Recurrent FSGS

The treatment is an unclear issue. Some authors show that there is no difference in the risk of recurrent FSGS between transplanted patients treated with standard doses of cyclosporine (CsA) or with azathioprine [119]. Instead, other investigators find that higher doses of CsA were associated with a lower risk [120]. The amelioration of proteinuria with cyclosporine may be related to the inhibitory action on the T cells and on production of their cytokines [121]. The anti-proteinuric effect may be related also to the inhibition of the dephosphorylation of synaptopodin that is determined by calcineurin, promoting the stabilization of the cytoskeleton in podocytes [122]. ACE-inhibitors and angiotensin-receptor blockers also improve the proteinuria in recurrent FSGS [123]. However, more commonly, plasmapheresis or immunoadsorption with protein A are used as therapy in recurrent FSGS. Best results seem to be achieved when plasmapheresis is started immediately when recurrence becomes clinically evident [74,88]. In cases of plasmapheresis and Rituximab resistance, Abatacept was effective in reducing proteinuria [124]. Savin et al. demonstrated that intravenous infusion of galactose reduced circulating permeability activity, probably thanks to a high affinity between galactose and permeability factors [125]. The monoclonal antibodies anti TNFα (Infliximab and Etanercept) significantly reduced proteinuria in a child with recurrent FSGS with relapse after discontinuation of anti-TNFα agent [126].

## 6. Membrano-Proliferative Glomerulonephritis (MPGN)

MPGN, also called mesangio capillary GN, is so defined because of the histological characteristics: mesangial matrix expansion and hypercellularity and the formation of a “double contour” resulting from the synthesis of new glomerular basement membrane with interposition of mesangial cells and leukocytes.

The traditional classification of MPGN was based on the location and type of electron dense deposits: type I, type II or Dense Deposit Disease (DDD) and type III [123]. Nevertheless, a new classification is based on the pathophysiology of MPGN and immunofluorescence studies at the biopsy: immune complex-mediated MPGN and complement mediated MPGN [127]. Immune complex-mediated MPGN typically results from viral (hepatitis C especially), bacterial, fungal, parasitic infections monoclonal gammopathy/dysproteinemias, or autoimmune disease (SLE, Sjogren’s syndrome, rheumatoid arthritis) [123]. Complement mediated MPGN, instead, results from dysregulation and overactivation of the alternative pathway of complement with glomerular deposition of C3 and other complement factors, and absent or poor immunoglobulin [128]. The term C3 glomerulopathy is also used to define complement mediated MPGN and on the basis of the electron microscopy may be subdivided into C3GN and DDD. In C3GN the deposits are often present in the mesangium and subendothelial region of the capillary walls, in DDD the deposits are large, extremely dense (osmiophilic), and intramembranous [129]. DDD generally has the higher rate of recurrence after transplantation.

Recurrent MPGN is seen in 27–65% cases of post-renal transplant resulting in graft loss in up to 50% of cases [130]. The recurrence rate of the second transplant seems to be even higher [131].

The pathogenesis of C3 glomerulopathy is related to uncontrolled activation of the alternate complement pathway as a result of aberrant gene mutations or acquired antibodies [123]. The most commonly acquired complement defect is the presence of C3 nephritic factor (C3NeF), an antibody which has the ability to block Complement Factor H (CFH) mediated decay by stabilizing C3 convertase [132]. By binding to C3 convertase, C3NeF leads to an overproduction of C3b, C5 convertase, and Membrane Attack Complex (MAC) [133]. Other complement abnormalities that have been identified as causes of C3 glomerulopathy include antibodies against factor B, CFH, and C3 convertase [134]. In addition, several genetic mutations have been reported, for example CFH, Factor I, membrane cofactor protein (MCP), and complement factor H related protein 5 (CFHR5) [135].

Risk factors for recurrence of C3 glomerulopathy after renal transplant are still poorly known. However, there is an association with the presence of monoclonal paraprotein [136], lower serum complement level [137], human leukocyte antigen B8, DR3, B49, and DR4 [123], higher proteinuria and the presence of crescents in the native kidney biopsy [138], instead C3NeFs levels seem to be not related to the risk of recurrence and the degree of disease activity [129].

Clinical presentation of recurrent C3 glomerulopathy includes proteinuria, hematuria, and higher serum creatinine, although DDD usually recurs later than C3GN and presents clinically only with allograft dysfunction [136]. However, patients with DDD commonly have low serum levels of C3 and C3NeF in circulation [132].

Strategies to prevent recurrence after transplantation are limited. Eculizumab, a monoclonal antibody that inhibits the formation of C5b-9 (MAC), binding C5 and consequently suppressing conversion of C5 to C5b by the C5 convertase [139,140,141], is used in patients with high risk of developing recurrent aHUS after kidney transplant [142]. However, there is no adequate evidence for its efficacy to prevent recurrent C3 glomerulopathy [133]. Despite this, several cases of patients with recurrent DDD successfully treated with Eculizumab were described in literature [143]. The treatment of C3 glomerulopathy in both the native and transplant kidney is uncertain [125]. The use of Cyclophosphamide and Mycophenolate mofetil may be advantageous in native disease, but their efficacy in recurrence is restricted [128]. In patients with C3 glomerulopathy due to genetic mutations in CFH, chronic infusions of fresh frozen plasma to replace absent complement factors may be useful [144]. The use of plasmapheresis and/or Rituximab in the treatment of recurrence due to pathogenic antibodies is a controversial topic in literature [128].

## 7. Hemolytic Uremic Syndrome (HUS)

HUS is a rare disorder characterized by thrombotic microangiopathy (TMA) that causes hemolytic anemia, thrombocytopenia, and acute renal failure [140,141,145,146]. HUS is usually classified in typical and atypical forms. Typical HUS is caused by Shiga toxin *Escherichia coli* producer infections [147]. Atypical HUS (aHUS) represents the 5–10% of all HUS and is characterized by an overactivation of complement with a dysregulation of the alternative pathway [148]. aHUS is characterized by a worse outcome than typical HUS. Previously renal transplantation was contraindicated in the patients with aHUS [149]. Indeed, rate of recurrent aHUS after renal transplantation is really significant, about 75–80% [150]. aHUS may be associated with genetic acquired or idiopathic forms. Mutations of the type “loss of function” have been identified, in genes that encode complement regulatory proteins such as complement factor H (CFH), complement factor I (CFI), membrane cofactor protein (CMP, CD46), and thrombomodulin (THBD) and mutation of the type “gain of function” in genes that encode C3 and complement factor B (CFB) [151].

The alternative complement pathway is physiologically and constantly activated with the spontaneous breakdown of C3 and the production of C3b which binds to CFB, that is hydrolyzed by complement factor D (CFD), thus leading to the formation of C3bBb (C3 convertase). Another molecule of C3b binds this complex and forms C5 convertase. Subsequently the complement pathway leads to form C5b-9 or MAC that is responsible for endothelial cell damage leading to micro-thrombosis [148] in different disease [152]. This pathway is usually regulated by numerous factors and abnormalities in these complement regulatory factors result in aHUS [153]. Furthermore, the rate of recurrent aHUS after renal transplantation is closely related to the specific mutated factor, membrane-bound or circulating [154]. Patients with mutation of membrane-bound factors, for example MCP, have an extremely low risk of developing recurrence, depending on donor genome [155]. On the other hand, patients with mutation of circulating factors, for example CFH and CFI, have a higher risk of developing recurrence leading to graft loss in 80–90% of cases. These factors are mainly produced by the liver; thus these abnormalities persist after kidney transplantation predisposing to recurrence [156]. Recipients with genetic mutation of CFH have a risk of about 80% to recur after transplantation [157]. This factor, competing with CFB for C3b binding, cleaves C3b acting as a cofactor for CFI, thus performs its decay accelerating activity (DAA) on C3 convertase [145]. Recipients with genetic mutation of MCP have a rate of risk about 20% to recur after kidney transplant [157]. MCP is a cofactor for the CFI-mediated inactivation of C3b and C4b and it is further expressed in the renal endothelium. Endothelial cells within kidney allograft express normal MCP, therefore recurrence in transplant patients is rare [158]. Recipients with genetic mutation of CFI have a rate of risk of about 90% to recur following isolated renal transplantation because it is mostly synthesized in the liver [157]. In addition, autoantibodies against the CFH have been associated with recurrent aHUS after renal transplant [159].

Multiple environmental factors may influence the recurrence of aHUS after renal transplantation: infections including cytomegalovirus, influenza virus, parvovirus B19, BK virus; the use of immunosuppressive drugs such as CNI and less frequently mTORi, rejection episodes [150,160].

Patients with post-transplant aHUS usually present with macroangiopathic hemolytic anemia, thrombocytopenia, and acute kidney injury, similar to non-kidney transplant patients. Typical laboratory abnormalities include an increased serum creatinine, evidence of hemolysis (such as increased reticulocyte count, schistocytes on peripheral smear, and increased serum lactate dehydrogenase), and a low platelet count. Histologic injuries on biopsy include vessel wall thickening (mainly arterioles or capillaries), intraluminal platelet thrombosis and obstruction of the vessel luminal, endothelial cell swelling and detachment from the basement membrane, glomerular ischemia, and onion-skin hypertrophy of the arteriolar walls. Differential diagnosis with acute antibody-mediated rejection is often difficult. Nevertheless, in the latter case C4d staining of peritubular capillaries and circulating donor specific antibodies are found [161].

Prevention of post-transplant recurrence includes the screening in the living-related donor to exclude genetic mutation [157]. Plasma therapy, including plasmapheresis, as a prophylactic treatment is still a topic of discussion in literature; instead, several data demonstrated the higher efficacy of prophylactic treatment with Eculizumab [142]. In patients with genetic mutations of circulating factors, produced mainly from liver, combined liver-kidney transplant may reduce the rate of recurrence [162]. Treatment of post-transplant aHUS recurrence with Eculizumab seems to be effective both as a first line therapy and as second line therapy for recipient’s refractory to plasma therapy [163]. In addition, recent studies have shown the efficacy of the use of either in combination [164].

## 8. Membranous Nephropathy (MN)

In literature there are few data available on recurrent MN, because *De novo* MN more frequently occurs in patients post-transplantation. The rate of recurrence in patients with idiopathic membranous GN following kidney transplantation is more than 40% and graft loss rates of over 10–15% at 10 years of follow-up have been reported, with a higher risk to recur in a second transplant [165].

The pathogenesis of MN is still unclear [166]. Antibodies directed against two types of proteins localized in podocyte have been identified: neutral endopeptidase (NEP) and M-type phospholipase A2 receptor (PLA2R). Approximately 70% of patients with idiopathic membranous nephropathy have shown to have circulating anti-PLA2R antibodies, noticeably IgG4 type [167]. Therefore, there is a direct relationship between the circulating levels of anti-PLA2R autoantibody and the risk of recurrence after kidney transplantation [168].

No other specific risk factors that may significantly affect the incidence of recurrence have been identified including living related donors, HLA epitopes, and a more aggressive disease in native kidney [169].

Clinically recurrent MN is characterized by proteinuria that can be in the nephrotic range [168]. Treatment of recurrence includes the use of corticosteroids, anti-proteinuric agents, alkylating agents, CNI, and Rituximab [170].

## 9. Secondary GN

Secondary GN, such as Pauci-Immune Crescentic GN, SLE, anti-GBM may recur later after renal transplantation and rarely lead to allograft failure.

Pauci-immune Crescentic GN is the most common cause of rapidly progressive glomerulonephritis, followed by anti-glomerular basement membrane (anti-GBM) disease and immune-complex glomerulonephritis and it is generally associated with circulating antineutrophil cytoplasmic antibodies (ANCA) [171]. Antineutrophil cytoplasmic antibodies (ANCA)-associated vasculitis (AAV) is characterized by necrotizing inflammation of small blood vessels and crescent formation [172]. Patients with ANCA associated vasculitis should be in clinical remission for at least 12 months, however, persistent ANCA positivity is not a contraindication to transplantation [171]. In patients with Pauci-Immune Crescentic GN rate of recurrence is about 17% and incidence of allograft loss is about 7.7% [173]. Therefore, thanks to modern post-transplant immunosuppression therapy, such as mycophenolate-mofetil and tacrolimus, rate of recurrence of these diseases is low, but regardless, these patients, particularly those with positive antiproteinase-3, require continuous monitoring. In both patients with native and transplanted kidney Rituximab may be a treatment of choice [171].

In patients with SLE rate of recurrence is about 30% and allograft loss is uncommon [174]. Clinical manifestation of recurrent lupus nephritic (LN) is generally modest proteinuria, microhematuria, cutaneous rash, and arthralgias [175]. Biopsy highlights in most cases mesangial lesions or atypical pauci-immune proliferative GN [176]. The risk factors associated with recurrent LN are black non-Hispanic ancestry, female gender, and young age. Patients with antiphospholipid (aPL) autoantibodies and those receiving the kidney from living donors also have a higher risk of recurrence [177]. The pathogenesis of this disease is multifactorial [178] and is related to innate and adaptive immune response, involving type I interferon signature as in antibody mediated rejection [179,180]. Recently, several studies have demonstrated the importance of signaling through type I transmembrane proteins, the Toll-like receptors (TLRs) associated with pathogen-associated molecular patterns (PAMPs) that are involved in defending against microbial infections but also in chronic inflammation and autoimmune disease such as SLE [181]. In addition, in these patients there is a decreased immune tolerance. The PAMPs may activate T-cells and, breaking tolerance, stimulate self-reactive B-cells that produce antibodies that react with cytoplasmatic and nuclear self-antigens, such as nephritogenic anti-double stranded (ds)DNA antibodies [182]. In patients with LN recurrence generally no change of therapy is necessary compared to the treatment used for the maintenance of the transplant [177]. However, patients with clinical manifestations and severe histopathologic lesions in the graft may require additional immunosuppressive treatment with bolus of steroid and higher doses of mycophenolate mofetil or cyclophosphamide intravenously in case of rapid renal deterioration with crescentic lesions and severe extra renal disease such as pulmonary hemorrhage and central nervous system involvement [183]. In patients with anti-GBM rate of recurrence is about 50% when circulating antibodies are still present before transplantation, instead if these antibodies are absent for at least 12 months recurrence is rare, but still possible [184]. However, when anti-GBM recurs the graft loss is rapid.

## 10. Conclusions

In the past recurrence was considered a minimal part of the causes of graft loss. However, at present the improvement of immunosuppressive therapy and long-term renal survival, by decreasing the incidence of acute rejection and indirectly through the consequent reduction of chronic allograft nephropathy, recurrent GN after renal transplantation is a significant contributor to late graft loss. The prevalence, epidemiology, risk factors, pathogenesis, clinical features, diagnosis, and treatment of recurrent GN are still unclear. Despite these difficulties, a careful analysis of the pathogenesis and underlying bimolecular mechanisms of both native and transplanted kidney diseases allows an adjustment of the therapy for each patient, thus optimizing renal transplant outcome.

## Abbreviations

ATG	Anti-thymocyte globulin
CALLA	Common Acute Lymphoblastic Leukemia Antigen
CNTFR	Ciliary neutrotrophic factor receptor
CR	Chronic rejection
DDD	Dense Deposit Disease
DSA	Donor-specific antibodies
ESKD	End-stage kidney disease
FSGS	Focal and segmental glomerulosclerosis
GBM	Glomerular basement membrane
Gd-IgA1	Galactose-deficient IgA1
GLEPP-1	Glomerular Epithelial Cell Protein-1
HLA	Human leukocyte antigen
HUS-TTP	Hemolytic uremic syndrome/thrombotic thrombocytopenic purpura
IgAN	IgA nephropathy
JAK/STAT	Janus kinase/signal transducer and activator of transcription
MAPK	Mitogen-activated protein kinase
MN	Membranous Nephropathy
MPGN	Membrano-proliferative GN
NEP	Neutral endopeptidase
PDGF	Platelet derived growth factor
PLA2R	Phospholipase A2 receptor
PTRO	Protein tyrosine phosphatase receptor type O
SLE	Systemic lupus erythematosus
suPAR	Soluble Urokinase-type Plasminogen Activator Receptor
TGF-β	Transforming growth factor
TNFSF13	Tumor necrosis factor superfamily 13
WT	Wilms’ tumor protein
